# Prescription patterns of anxiolytics in remote consultation versus in-person consultation: a cross-sectional study in French general practice

**DOI:** 10.3399/BJGPO.2024.0176

**Published:** 2025-09-10

**Authors:** Flora Descans, Vincent Tarazona, David De Bandt

**Affiliations:** 1 General Practice Department, University of Versailles – Saint Quentin en Yvelines, Paris, France; 2 Center for Research in Epidemiology and Population Health, The French National Institute of Health and Medical Research, Villejuif, France

**Keywords:** remote consultation, anxiolytics, anti-anxiety agents, general practice

## Abstract

**Background:**

Teleconsultation is a new mode of medical consultation in France, which is poorly evaluated, and anxiolytics are among the drug classes at risk of over-medication.

**Aim:**

To analyse the prescription patterns of anxiolytics in remote consultation (RC) versus in-person consultation (IPC) in general practice.

**Design & setting:**

This is a retrospective cross-sectional study in French general practice in 2021.

**Method:**

Consultations at volunteer general practice offices were analysed. The mode of consultation was extracted. For each consultation with an anxiolytic prescription, the prescribed daily dose (PDD), the age, sex, initiation or renewal of treatments, and the length time before the next consultation were recorded. χ^2^ was performed to compare the correlation between the mode of consultation and the drug prescription. Then, for consultation with anxiolytic prescription, multivariate models were conducted to analyse the PDD and the average time interval before the next consultation adjusted for consultation characteristics.

**Results:**

A total of 46 880 consultations were included from 11 GPs. The rate of consultations with anxiolytic prescriptions was identical in RC and IPC, but the PDD was higher by 6.17 in RC compared to IPC (95% confidence interval [CI] = 0.21 to 12.12; *P* = 0.04). The average time interval before the next consultation was identical in RC and IPC.

**Conclusion:**

The rate of consultations with anxiolytic prescriptions is identical in RC and IPC, but the prescribed doses are higher in RC. Given the adverse effects of these treatments, GPs should reflect on their prescriptions to avoid over-medication.

## How this fits in

Remote consultation (RC) has become a part of general medical care in France since 2020 and the COVID-19 pandemic. RC raises questions about the risk of over-medication, and anxiolytics are noted as being at risk of over-medication. This study compares the prescription of anxiolytics in teleconsultation with in-person consultations (IPC). This study finds a similar rate of consultations with anxiolytic prescriptions in both teleconsultation and face-to-face consultations, but the prescribed doses are higher in RC.

## Introduction

Since 15 September 2018, GPs in France have been able to offer remote consultation (RC) services, and patients covered by French public health insurance are eligible for reimbursement. Patients must comply with the coordinated care pathway and have had an in-person consultation (IPC) with their referring doctor within the preceding 12 months.^1^ RCs are promoted as a quick and convenient way to access medical expertise, offering a partial solution to the challenges posed by healthcare provider shortages.

In 2019, 230 million consultations were conducted by GPs, but only 80 000 of these were RCs.^2^ During the COVID-19 pandemic, exemptions to reimbursement conditions facilitated the expansion of RC usage including the removal of the coordinated care pathway requirement and full reimbursement for all patients until 30 September 2022.^3^ As a result, the number of RCs increased significantly to 13.5 million in 2020, stabilising to 9.4 million in 2021, which is approximately 5% of total general practice consultations per year.

Despite its benefice, RC have major limitations including the lack of physical examinations, and the potential disruptions to the doctor–patient relationship.^4^ As a relatively recent practice, data on the coherence of prescriptions with clinical symptoms and medical guidelines remain limited. A 2021 report by the French National Academy of Medicine emphasised the importance of identifying differences between RC and IPC.^5^ Previous studies have highlighted concerns such as over-medication of antibiotics and sick leave during RC, prompting health authorities to impose restrictions on prescriptions in RC settings.^6,7^

In addition to accelerating the democratisation of teleconsultations, the COVID-19 pandemic significantly impacted the prevalence of depression and anxiety in France. Factors such as social isolation, fear of infection, and financial difficulties contributed to this rise.^8^ The World Health Organization (WHO) reported a 25% increase in cases of anxiety and depression during the first year of the pandemic.^9^ Consequently, the consumption of antidepressants in France increased after the onset of COVID-19, and the use of anxiolytics peaked during the initial lockdown in 2020.^8^

Anxiolytics are prescribed in primary care for a short-term use for anxiety crises, as well as for panic disorders or generalised anxiety disorders. The most well-known are benzodiazepines and related substances that can cause side effects and lead to addiction. Despite recommendations from health authorities, France remains one of the highest consumers of these medications worldwide, and benzodiazepines are recognised as medications at high risk of overuse. In 2015, approximately 13% of French people reported using a benzodiazepine. GPs are the primary prescribers.^5,10–12^ Studying anxiolytic prescriptions in RCs is particularly important, given the high rate of usage in France, the potential for serious adverse effects, and the risk of over-medication that RC may exacerbate.

The main objective of this study was to compare GPs’ anxiolytic prescribing rate in IPC versus RC. Secondary objectives were to identify factors that might influence the prescription of anxiolytics, according to the type of consultation in patients with anxiety disorders treated with anxiolytics. This study also compared the time to the next consultation following either IPC or a RC for patients treated with anxiolytics. Our aim was to document the risk-benefit balance of using RC in anxiety disorders, particularly regarding the potential for over-medication, and to gain a clearer understanding of the factors that influence anxiolytic prescriptions.

## Method

### Study design

We conducted a retrospective cross-sectional study in general practice in 2021, in accordance with Strengthening the Reporting of Observational studies in Epidemiology (STROBE) checklist guidelines to ensure high reporting quality.^13^

Patient data were anonymised and extracted from medical software by data protection officers (DPO) in volunteer general practices.^14^ Statistical analyses were performed using Rstudio (version 12.0).

### Recruitment

GPs in the Ile-de-France region using the Weda medical software were eligible to participate. Weda is the only French medical software for GPs with a health policy quality certificate and equipped with an automated data extraction system.^15^ Only physicians with general practice as principal activity were recruited. Given the methodological challenges in recruiting busy GPs, who are often their own DPO responsible for sending anonymised data, we employed a homogenous snowball sampling approach often used in qualitative research to expand the pool of participants.^16^ We began by recruiting initial volunteer physicians from the Weda database who then referred us to additional GPs. Recruitment ceased once the required number of consultations had been reached.

### Data extraction

Consultation billing codes were used to distinguish IPC from RC. Screen-based billing codes were classified as RC, while face-to-face codes were classified as IPC. Consultations with unidentifiable billing codes or with restricted patient access were excluded.

Anxiolytics prescriptions were identified by Anatomical Therapeutic Chemical (ATC) classification class N05B, and other benzodiazepine receptor agonists by ATC class N05CD and N05CF. For each consultation resulting in an anxiolytic prescription, we recorded the patient’s age, sex, prescribed molecule and dose, and the time until the next consultation. We also noted the type of prescription, whether it was a first prescription or a renewal within the previous 12 months.

To quantify prescriptions, the total anxiolytic dose prescribed per consultation was divided by the WHO defined daily dose (DDD) for each class prescribed molecule.^17^ This enabled us to determine the prescribed daily doses (PDD), representing the total dose prescribed per consultation.

### Statistical analysis

The primary outcomes were the occurrence of consultation with anxiolytic prescription in RC or IPC and the quantity of drug prescribed, expressed in PDD. The secondary outcomes was the number of days until the next consultation after an anxiolytic prescription in IPC versus RC.

According to the literature, 13% of French people used a benzodiazepine in 2015. Based on this, we hypothesised that 13% of consultations would result in an anxiolytic prescription. Assuming approximately 230 million GP consultations in 2021, including 5% of RCs, we estimated that 19 000 total consultations, including 950 RCs, would be required to detect a 5% statistical difference with 90% power.^2,5^

The analysis of the primary outcome was performed using a χ^2^ test to compare the rate of RC with anxiolytic prescriptions and the rate of IPC with anxiolytic prescription. Group differences in patient sex and prescription type (initiation versus renewal) were also analysed using χ^2^ tests. Parametric univariate Student’s *t*-tests were conducted to compare mean PDD per consultation, mean age, and mean interval to the next consultation between IPC and RC groups.

Mean anxiolytic PDD was analysed using a multiple logistic regression model adjusted for sex, age, type of prescription, and the type of consultation (IPC or RC). Time to next consultation was analysed using a multiple linear regression model adjusted for age, sex, type of prescription, and the mode of consultation.

## Results

Eleven doctors from the Ile-de-France region participated in this study, including six established practitioners and five substitutes. Their practices were situated in urban or semi-urban areas and were organised in diverse ways, including multidisciplinary health centres, group medical practices, and individual offices. Four of the established practitioners also served as university intern supervisors. The average age of all participating doctors was 38 years (Table 1).

**Table 1.** Characteristics of included volunteer physicians

Attributes	Type of attribute	*n* = **11**
**Age, years**	≤30	3
	31–40	5
	41–50	2
	≥60	1
	Mean age = 38	
**Sex**	Male	5
	Female	6
**Office**	Alone	1
	MHC	6
	GPG	4
**Grade**	Titularised	6
	Replacement	5
**Activities**	Urban	4
	Semi-urban	7
**Teaching activities**	Yes	4
	No	7

Alone = a physician working alone in their office. MHC = multidisciplinary healthcare centre in primary care with at least 2 GPs and a nurse. GPG = general practice group with GPs only. Titularised = a physician who permanently holds and is fully responsible for their own practice. Replacement = a physician who temporarily covers another (titularised) physician’s practice during their absence. Urban = a physician whose practice is conducted exclusively in an urban setting. Semi-urban = a physician whose practice is divided between urban and rural (countryside) settings

**Figure 1.**
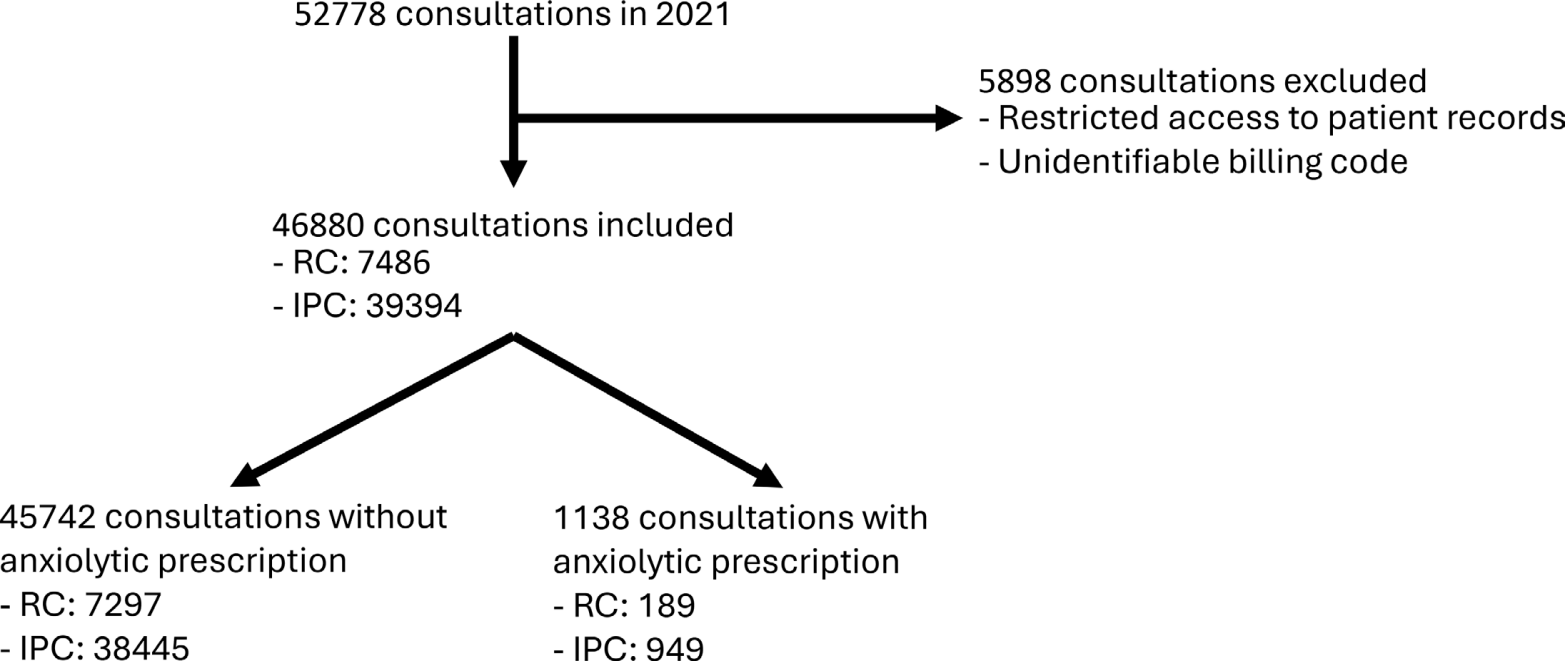
Flowchart identifying consultations with anxiolytic prescriptions

In 2021, the participating doctors conducted 52  778 consultations. In total, 5898 consultations were excluded owing to unidentifiable billing codes or restricted access to patient records, leaving 46 880 consultations for inclusion in the study. Of these, 39 395 (84.03%) were IPC and 7486 (15.97%) were RC. Of the total consultation included in the study, 1138 (2.43%) resulted in a prescription of anxiolytics, including 949 IPC and 189 RC (Figure 1). The average age of patients was 58.49 for IPC and 52.33 for RC. Females consulted 2.26 times more often than males for all consultations, representing 68.81% of IPC and 71.96% of RC. The average prescribed dose of anxiolytics was 26.22 PDD for all consultations, representing 25.38 for IPC and 30.46 for RC. The average time to the next consultation was 57.84 days for IPC and 54.14 for RC (Table 2). The Supplementary appendix provides a list of the anxiolytic molecules prescribed by GPs in our sample. Some of these molecules were not prescribed frequently enough to allow meaningful statistical analysis.

**Table 2.** Univariate analyses comparing the characteristics of in-person consultations and remote consultation

All consultation *n* = **46 880**	IPC		RC		Statistic		
	No anxiolytic	Anxiolytic	No anxiolytic	Anxiolytic	OR	95% CI	*P-value*
Anxiolytics	38 445	949	7297	189	1.05	0.90 to 1.23	0.55
**Consultation with anxiolytics prescription *n* = 1138**	**IPC**		**RC**		**Statistic**		
	Male	Female	Male	Female	OR	95% CI	*P-value*
Sex	296	653	53	136	0.86	0.61 to 1.21	0.44
	Initiation	Renewed	Initiation	Renewed	OR	95% CI	*P*-value
Prescription	208	738	34	153	0.79	0.53 to 1.18	0.28
	Mean	*n*	Mean	*n*	Coefficient	95% CI	*P-value*
Age, year	58.49	948	52.33	189	-6.15	–9.07 to –3.24	<0.01
Consultation interval, day	57.84	894	54.14	174	-3.71	–13.72 to 6.30	0.47
Prescribed daily dose	25.38	949	30.46	189	5.08	–0.84 to 11.01	0.09

χ^2^ test was performed for anxiolytics, sex, and prescription. Parametric *t*-test was performed for age, consultation interval, and PDD. IPC = in-person consultation. *n* = number of observations. OR = odd ratio. *P* = probability value. PDD = prescribed daily dose. RC = remote consultation

A total of 46 880 consultations were analysed, and the rate of IPC with anxiolytic prescriptions was found to be non-significantly different from the rate of RC with anxiolytic prescriptions (2.41% of IPC versus 2.52% of RC; OR = 1.05; 95% CI = 0.90 to 1.23). For those who received anxiolytic prescriptions, univariate analyses indicated that the average age of patients was significantly lower in RC than in IPC with a mean difference of –6.15 years (95% CI = −9.07 to –3.24; *P*<0.01). No significant differences were observed in terms of sex, type of prescription, the average time to the next consultation, or average PDD between RC and IPC.

Multivariate analyses of consultations with anxiolytic prescriptions (Table 3) found an average increase in PDD of +6.17 in RC compared with IPC (95% CI = 0.21 to 12.12; *P* = 0.04). An increase of+0.20 PDD is observed for each additional year of patient age (95% CI = 0.08 to 0.32; *P*<0.01). In contrast, the PDD significantly decreased by an average of -9.99 when patients were prescribed an anxiolytic treatment for the first time (95% CI =−15.48 to -4.49; *P*<0.01). The study reveals no statistically significant difference in PDD between males and females.

**Table 3.** A: Multivariate logistic regression of PDD adjusted on the type of consultation, patient sex, type of prescription, patient age. B: Multivariate logistic regression of interval between the next consultation adjusted on the type of consultation, patient sex, type of prescription, patient age and PDD

Consultation with anxiolytics prescription *n* = **1138**			
Prescribe daily dose	**Coefficient**	**95% CI**	***P*-value**
IPC (Ref) versus RC	6.17	0.21 to 12.12	0.04
Female (Ref) versus male	1.81	–2.94 to 6.56	0.46
Renewed (Ref) versus initiation	–9.99	–15.48 to –4.49	<0.01
Age	0.20	0.08 to 0.32	<0.01
Consultation interval (day)	**Coefficient**	**95% CI**	***P*-value**
IPC (Ref) versus RC	–0.23	–10.65 to 9.87	0.96
Female (Ref) versus male	8.05	0.03 to 16.07	0.05
Renewed (Ref) versus initiation	3.88	–5.72 to 13.48	0.43
Age	0.36	0.16 to 0.57	<0.01
Prescribe daily dose	–0.11	–0.21 to –0.01	0.02

IPC = in-person consultation. *n* = number of observations. PDD = prescribed daily dose. ref = reference categories for analyses. RC = remote consultation

The multivariate analysis of the average time interval before the next consultation following a consultation with anxiolytic prescription (Table 3), adjusted for type of consultation, sex, age, type of prescription, found no statistical difference between IPC and RC. This interval decreased by –0.11 days for each additional PDD prescribed during the consultation (95% CI =−0.21 to –0.01; *P* = 0.02). In contrast, the time interval increases of 0.36 days per additional year of patient age (95% CI = 0.16 to 0.57; *P*<0.01) and by 8.05 days for males compared with females (95% CI = 0.03 to 16.07; *P* = 0.05). The initial prescription was not associated with a modified time interval compared with a renewal of anxiolytic prescription.

## Discussion

### Summary

Consultation rates involving anxiolytic prescriptions in IPC and RC are comparable, but the prescribed anxiolytic dose is 6.17 PDD higher in RC. Female patients consult their doctor 2.26 times more often than males for consultations in which anxiolytics are prescribed, but the rate of RC use and the dose prescribed do not differ between the sexes. RC use is associated with a younger patient age with a mean of 6.15 years lower than in IPC, and with higher prescribed doses in older patients, which increase by an average of 0.20 PDD per additional year. The rate of consultation with initial anxiolytic prescriptions is similar between IPC and RC, but the dose prescribed at initiation is on average 9.99 PDD lower compared with treatment renewals.

During consultations involving anxiolytic prescriptions, the average time intervals before the next consultation does not differ significantly between RC and IPC. Females return for consultation on average 8.05 days sooner than males, while older patients take longer to return with an average delay of 0.36 days per additional year of age. Additionally, higher prescribed anxiolytic doses are associated with shorter intervals between consultations, decreasing by an average of 0.11 days per additional PDD.

### Comparison with existing literature

GPs prescribe anxiolytics as frequently in RC as in IPC, but at a significantly higher doses in RC (p = 0.04). These results align with earlier preliminary analyses, indicating a similar consultation rate of consultations for psychiatric reasons in IPC and RC.^18^ The higher dose prescribed in RC could be related to differences in the clinical or sociological characteristics of patients or to physician prescribing habits compared with IPC.^4,19^ An altered doctor–patient relationship in RC, crucial in managing anxiety disorders, may contribute to these results. It can be more difficult to interpret nonverbal cues during RC than IPC, leading to different physician assessment of symptom intensity.^4,20^ This alteration may bias the physician’s perception of a patient’s expectations and increase clinical uncertainty, promoting higher medication dosage.^21^ It is also possible that during the COVID-19 pandemic, patients who chose RC for fear of contamination had more anxiety symptoms, thus requiring higher anxiolytic doses.

The male-to-female ratio for anxiolytic prescriptions is similar in IPC and RC and the dose prescribed does not differ between males and females. The number of consultations with anxiolytic prescriptions is higher for females. This aligns with existing data showing that women generally consume more healthcare services and use more anxiolytics than men.^5,22^ This difference could be related to the higher mental load typically reported in women.^12,23^

The average patient age for anxiolytic prescription in RC is lower than that in IPC. In France in 2021, 45.2% of general medicine RC involved patients aged 15–44 years, compared with 28.7% of IPC.^2,18^ Younger patients are more inclined to use RC partly because they have better access to digital tools. Our finding that prescribed anxiolytic doses increases with age is consistent with previously published studies showing that older patients are the highest consumers of anxiolytics.^24^

No difference was found in the rate of anxiolytic treatment initiation between IPC and RC, but the prescribed doses are higher for renewal than for initiation. These findings are in line with clinical guidelines recommending introduction of the smallest effective dose to limit treatment duration.^10^ This result may also reflect a tolerance effect of anxiolytics, where dose prescribed increase over the course of treatment renewals. Further studies could clarify the mechanisms behind anxiolytic treatment renewals.

The average time before the next consultation following an anxiolytic prescription is not shorter in RC than in IPC. Females return sooner than males, consistent with other studies showing no significant difference between RC and IPC intervals for psychiatric reasons but a higher healthcare use among women. Such results could also be related to a higher mental load in women.^5,12,18,22,23^

Contrary to previous finding suggesting shorter time intervals between consultations in older patients, our data indicate that consultation intervals increase with age after anxiolytic prescription.^18^ One explanation could be that older patients are more often prescribed anxiolytics for long-term use and in particular for hypnotic effects, whereas younger patients more commonly receive short-term prescription for acute anxiety disorders, resulting in more frequent renewals.^25–28^ This also explains why higher PDD is correlated with shorter intervals before the next consultation. Shorter duration prescriptions are recommended by clinical guidelines for anxiety and require more frequent renewals.^10^ Numerous campaigns promoting gradual dose reduction have been initiated to decrease the use of hypnotics, especially among older patients who are the highest consumers.^29^

### Strengths and limitations

The main strength of this study is the novelty of the research topic. To date, no published study in France has analysed anxiolytics prescribing in RC. The required number of consultations to include was reached, and data collection covers the entire year of 2021, reducing the seasonal influences on the results and increasing the statistical power. Consultation characteristics align with literature. RC users tend to be younger, and women are more frequently prescribed anxiolytics than men.^2,5,12,22^

Several factors limit the strength of this study. The main limitation is the small number of volunteer physicians included, which restricts the representativeness of the participants and constrains the generalisability of our findings. The average age of recruited volunteer doctors is younger than the national average, and the RC rate is higher than the French national average in 2021, likely because younger urban doctors adopt RC more frequently than their colleagues.^2^ Participating doctors primarily practice in the Île-de-France region, which may not represent the entire population of French GPs. Although, data were collected during the COVID-19 pandemic, which may have increased the rate of RC utilisation, the rate of RC in 2021 is like the years after the COVID-19 pandemic.^30^ Lastly, this study analysed anxiolytic prescriptions by practitioners rather than actual patient anxiolytic consumption. Future interventional studies could clarify whether prescription patterns correspond directly to patient consumption in RC versus IPC.

### Implications for research and practice

The rate of consultations with anxiolytic prescriptions is similar in IPC and RC, but the prescribed dose is higher by 6.17 PDD in RC. These findings are likely related to the alteration of the doctor–patient relationship inherent in RC and the absence of clinical examination, which may have increased clinician uncertainty and encourage over-medication. Additional research focused on GPs’ perceptions of RC in managing anxiety disorders could shed more light on the mechanisms leading to higher prescribed doses of anxiolytics. GPs should question their prescribing practices in RC to limit the risks of over-medicalisation. This is an urgent need given the growing discussion around prescription restrictions, including sick leave, in RC. Questioning and promoting appropriate prescribing behaviours will help to ensure the safety of anxiolytic prescriptions, whether administered in-person or remotely.
